# Impact of dietary gut microbial metabolites on the epigenome

**DOI:** 10.1098/rstb.2017.0359

**Published:** 2018-04-23

**Authors:** Clarissa Gerhauser

**Affiliations:** Epigenomics and Cancer Risk Factors, German Cancer Research Center (DKFZ), Heidelberg, Germany

**Keywords:** epigenomics, gut microbiota, metabolism, diet, human health

## Abstract

Within the past decade, epigenetic mechanisms and their modulation by natural products have gained increasing interest. Dietary bioactive compounds from various sources, including green tea, soya, fruit and berries, cruciferous vegetables, whole grain foods, fish and others, have been shown to target enzymes involved in epigenetic gene regulation, including DNA methyltransferases, histone acetyltransferases, deacetylases and demethylases *in vitro* and in cell culture. Also, many dietary agents were shown to alter miRNA expression. *In vivo* studies in animal models and humans are still limited. Recent research has indicated that the gut microbiota and gut microbial metabolites might be important mediators of diet–epigenome interactions. Inter-individual differences in the gut microbiome might affect release, metabolism and bioavailability of dietary agents and explain variability in response to intervention in human studies. Only a few microbial metabolites, including folate, phenolic acids, *S*-(−)equol, urolithins, isothiocyanates, and short- and long-chain fatty acids have been tested with respect to their potential to influence epigenetic mechanisms. Considering that a complex mixture of intermediary and microbial metabolites is present in human circulation, a more systematic interdisciplinary investigation of nutri-epigenetic activities and their impact on human health is called for.

This article is part of a discussion meeting issue ‘Frontiers in epigenetic chemical biology’.

## 1. Introduction

### (a) The gut microbiome: our second genome

Studies have identified large inter-individual differences in gut microbial composition, with consequences for human health [1,2]. A recent large-scale sequencing programme of 124 individuals identified about 3.3 million non-redundant microbial genes, derived from 576.7 gigabases of sequence [3]. Thus, the gut metagenome (the collective genetic information derived directly from a faecal sample by deep sequencing) is about 150 times larger than the human genome. Eighteen bacterial species were detected in all individuals, 57 in greater than or equal to 90% and 75 in greater than or equal to 50% of individuals [3]. A following study revealed that based on their predominant gut bacterial communities, individuals could be grouped into three main clusters or enterotypes, namely *Bacteroides*, *Prevotella* and *Ruminococcus*. Host properties such as age, body mass index or gender did not explain the enterotypes. Rather, enterotypes seem to differ in their choice of energy source [4]. Wu *et al*. [5] postulated that enterotypes were strongly associated with long-term diets, particularly protein and animal fat (*Bacteroides*) versus plant-derived carbohydrates and fibre (*Prevotella*). Subsequent studies questioned the existence of distinct enterotypes [6] and instead proposed that microbial gene richness (the number of microbial genes per individual) might be more relevant for human health status [7,8]. Populations could be separated by a bimodal distribution of gene counts. Low gene richness was related to overall adiposity, insulin resistance, dyslipidaemia and an inflammatory phenotype compared with the high gene group [7,8]. In two large metagenomics analyses of Dutch and Belgian populations, low microbial diversity has been linked to consumption of high-fat whole milk, sugar-sweetened drinks, higher total energy and carbohydrate intake, and snacking, whereas high microbial diversity has been associated with consumption of coffee, tea, red wine and dark chocolate as sources of polyphenols [9,10]. Overall, these studies indicate that the metagenomic composition can be modified by (long-term) dietary patterns [1,2].

### (b) Regulation of the epigenome

The term ‘epigenetics’ refers to modifications in gene expression caused by heritable, but potentially reversible, changes in DNA methylation and chromatin structure. Major epigenetic mechanisms include DNA hyper- and hypomethylation [11], remodelling of the chromatin, modification of histones by histone acetylation and methylation (among others), and noncoding RNAs [12].

The DNA methyltransferase (DNMTs) family of enzymes catalyses the transfer of methyl groups from *S*-adenosyl-l-methionine (SAM) to DNA. In mammals, this occurs at the 5-position of cytosine (C) in the context of CpG dinucleotides, generating 5-methylcytosine (5mC). CpG dinucleotides are not evenly distributed in the genome: CpG-dense regions (CpG islands or CGIs) are located in the promoter regions of genes and are mostly unmethylated in healthy tissues, allowing active gene transcription. Conversely, intra- and intergenic regions have lower CpG density and are highly methylated, thus limiting DNA accessibility and maintaining genomic stability. Focal gain in methylation at CGIs in promoter regions, for example, of tumour suppressor genes, concomitant with global loss of methylation (hypomethylation), especially at repetitive sequences, is thought to be involved in the aetiology of cancer. In contrast to irreversible genetic alterations (mutations, deletions, etc.), genes silenced by epigenetic modifications are still intact and can be reactivated [13,14].

Chromatin accessibility and gene expression is controlled by various post-translational modifications of N-terminal histone tails, including acetylation, methylation, phosphorylation, ubiquitinylation, sumoylation and ADP ribosylation [15,16]. Acetylation of histone tails by histone acetyltransferases (HATs) opens up the chromatin structure, allowing transcription factors to access the DNA [17]. Histone acetylation is reversed by histone deacetylases (HDACs) that remove histone acetyl groups by catalysing their transfer to coenzyme A (CoA), leading to chromatin condensation and transcriptional repression. Beside the currently known HDACs 1–11, structurally unrelated sirtuins (SIRTs) possess deacetylating activity, using NAD^+^ as a cofactor [18]. Histone lysine methylation has activating or repressive effects on gene expression, dependent on the lysine residue that is modified by methylation and the number of methyl groups [15]. More than 30 histone methyltransferases have been identified in humans that transfer methyl groups from SAM to lysine residues [19–21]. Histone methylation marks are removed by histone lysine demethylases (HDMs), for example, by lysine-specific demethylase 1 (LSD1) and the family of about 20 Jumonji domain-containing (JmjC) histone demethylases [22].

Noncoding (nc) RNAs also possess a regulatory effect on gene expression. MicroRNAs (miRNAs) are small ncRNAs of 20–22 nt that inhibit gene expression at the posttranscriptional level either by imperfect base-pairing to the mRNA 3′-untranslated regions to repress protein synthesis, or by affecting mRNA stability (reviewed in [23]). Identification and functional evaluation of long noncoding (lnc) RNAs (greater than 200 nt) has become an additional area of scientific interest [24,25].

## 2. Impact of microbial metabolites on the epigenome

Dietary agents from various sources, including green tea, fruit and berries, cruciferous vegetables and soya, directly target enzymatic activities or modulate expression of enzymes involved in epigenetic gene regulation. Therefore, they might affect numerous biological mechanisms, including signal transduction mediated by nuclear receptors and transcription factors such as NF-κB, cell proliferation and cell-cycle progression, cellular differentiation, DNA repair, apoptosis induction, cell motility, metastasis formation and cellular senescence (reviewed in [26–28]). Recent research has indicated that the gut microbiota and gut microbial metabolites might be important mediators of the diet–epigenome interaction (previously reviewed in [29–31]). The present overview is intended to summarize recent evidence from *in vitro* analyses (table 1) and *in vivo* investigations in rodent models and human intervention studies (table 2).

**Table 1.** Overview of dietary agents and microbial metabolites affecting the epigenome. ACL, ATP-citrate lyase; DHA, docosahexaenoic acid; EC, epicatechin; PCG, polycomb group; SCFA, short chain fatty acid; TNFα, tumour necrosis factor α.

class of compounds	effect of microflora	microbial metabolite	epigenetic mechanisms of metabolites and *in vitro* activity	reference
**folic acid, B-vitamins** 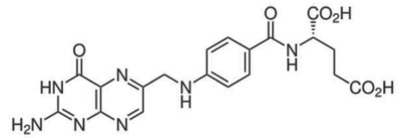	synthesis of folic acid from pteridine precursors, folate deficiency after antibiotic use		involved in 1-carbon metabolism and generation of *S*-adenosyl methionine (SAM); altered SAM levels influence methylation reactions of DNA and histones	[32–34], review in [35]
**soya isoflavones**, daidzein 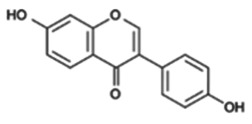	metabolization to *S*-(−)equol with ↑ bioavailability ↑ oestrogenicity	*S*-(−)equol 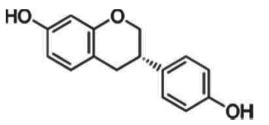	inhibition of DNA methylation, modulation of histone modifications, up- and downregulation of ncRNAs	review in [28,36]
**flavonoids**, e.g. apigenin 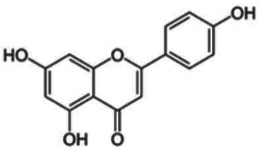	reductive cleavage and hydrolysis to hydroxyphenylacetic acids levels in human faecal water: 0–0.44 mM [37]	e.g. 3-(4-hydroxyphenyl) propionic acid 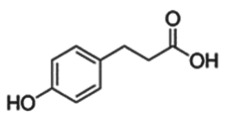	— 20–88% inhibition of DNMT activity at 20 and 40 µM — inhibition of HDAC activity with IC_50_ 0.19–5.47 mM	[38–41]
**green tea catechins (GTCs)**	reductive cleavage, and dehydroxylation to phenolic acids	e.g. 4-hydroxy-5-(3,4-dihydroxyphenyl)valeric acid from EC		[36,38]
**ellagic acid and ellagitannins** ellagic acid, e.g. in black raspberries 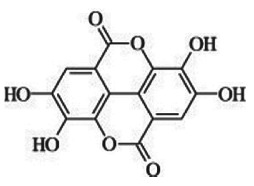 punicalagin from pomegranate 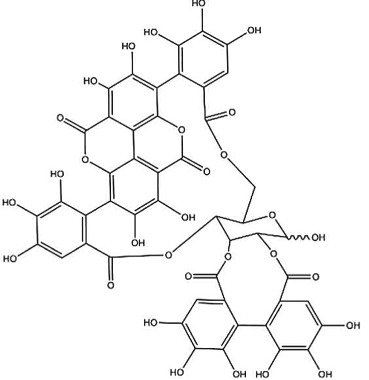	sequential decarboxylation and dehydroxylation to urolithin (URO) A, B or C plasma concentrations: URO-A glucuronide 0.024–35 µM URO-B glucuronide 0.012–7.3 µM urine levels up to 100 µM	urolithin A 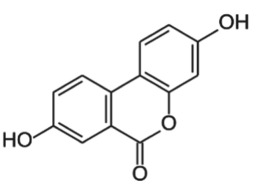 urolithin B 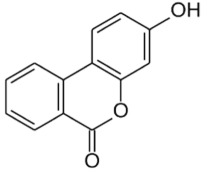 urolithin C 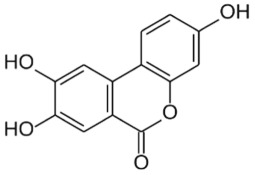	— ellagitannin BJA 3121 (50 µg ml^−1^, 6 h) alters expression of 25 miRNAs in HepG2 hepatoma cells — ellagitannin metabolites or mixtures (100 µM, 48 h) downregulate onco-miR-244 in Caco-2 and upregulated tumour suppressor miR-215 in HT-29 colon cancer cells — ellagic acid (5 µM, 24 h) prevents TNFα-mediated reduction of HDAC activity in THP-1 human monocytes; no direct effect on HDAC activity — ellagic acid and urolithins (5 µM, 24 h) inhibit TNFα-mediated induction of HAT activity in THP-1 human monocytes; only weak direct inhibition of HAT activity by ellagic acid	review in [42,43] [44] [45] [46]
**cruciferous vegetables** glucosinolates, e.g. glucoraphanin in broccoli 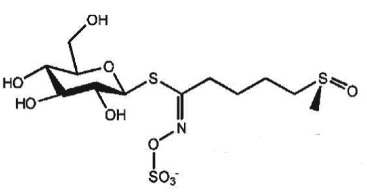	deglucosylation by plant or microbial thioglycosidases, intramolecular Loessen rearrangement to isothiocyanates and other compounds	e.g. sulforaphane (SFN) 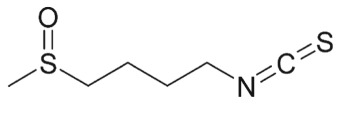	cysteine metabolite of SFN inhibits HDAC activity at 9 and 15 µM	[47]
**dietary fibre**	fermentation to SCFAs acetate, propionate, butyrate	e.g. butyrate 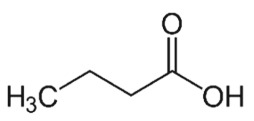	— inhibition of HDAC activity at mM concentrations — substrate for ACL to generate acetyl-CoA and stimulation of histone acetylation by HATs at sub-mM concentrations	[48]
**long-chain omega 3 fatty acids,** e.g. α-linolenic acid (ALA) 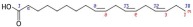	gut microbiota can metabolize, e.g. ALA to eicosapentaenoic acid (EPA)	EPA 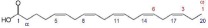 DHA 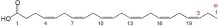	— treatment of breast cancer cell lines with EPA and DHA (40 and 80 µM) for 3–8 h. — dose-dependent reduced expression of the PCG protein EZH2 due to proteasomal degradation — reduced H3K27me3 levels	[49]

**Table 2.** *In vivo* rodent and human investigations linking microbial metabolites and epigenetic mechanisms. ACM, azoxymethane; ALA, α-linolenic acid; CRC, colorectal cancer; EC, epicatechin; EPA, eicosapentaenoic acid; FAP, familial adenomatous polyposis; PBMC, peripheral blood mononuclear cell; SCFC, short chain fatty acid.

class of compounds	study design	major outcome	ref.
**green tea catechins (GTCs)** EC, epicatechingallate (ECG), epigallocatechin (EGC), epigallocatechingallate (EGCG), high molecular weight tannins	TRAMP mouse model for prostate cancer — wild-type mice (*n* = 10–20 per group) receiving 0 or 0.3% green tea extract in drinking water — TRAMP mice (*n* = 20–40) receiving 0 or 0.3% green tea extract in drinking water for eight or 20 weeks — dose–response study in 5–10 TRAMP mice per group with 0.1, 0.3, or 0.6% green tea extract in drinking water for 12 weeks	no effect on tumour formation and genome-wide DNA methylation in prostate, liver and gut	[50]
**black raspberries (BRBs)** ellagic acid and ellagitannins, quercetin glycosides, anthocyanins	Phase I human intervention study with 20 colon cancer patients, daily dose 60 g of BRB powder for four to nine weeks	↓ DNMT expression in tumour tissue; promoter demethylation of WNT-signalling genes	[51]
black raspberries (BRBs)	Phase Ib human intervention study with 7 FAP patients receiving BRB powder (60 g) orally plus two BRB suppositories (720 mg) versus with 7 FAP patients receiving placebo plus two BRB suppositories for nine months	— burden of rectal polyps decreases in both groups — no additional effect of oral BRBs — BRBs significantly decrease cellular proliferation, DNMT1 protein expression, and p16 promoter methylation in rectal polyps (adenomas) from responders but not from non-responders — no effect on promoter methylation of SFRP2 and WIF1	[52]
black raspberries (BRBs)	*Apc*^*Min*/+^ mouse model for colon cancer — wild-type C57BL/6 J+/+ mice on control diet (*n* = 20), *Apc*^*Min*/+^ mice on control diet (*n* = 19) — *Apc*^*Min*/+^ on control diet supplemented with 5% BRBs (*n* = 24) for eight weeks	— BRB intervention significantly decreases intestinal and colonic polyp number and size in *Apc*^*Min*/+^ mice — BRBs reverse expression of 23 *Apc*-regulated metabolites in colonic mucosa, liver and faeces, e.g. putrescine and linolenate — epigenetic mechanisms not investigated	[53]
black raspberries (BRBs)	metabolomic study in mice — wild-type C57BL/6 J+/+ mice on control diet (*n* = 7) — or on control diet supplemented with 5% BRBs (n = 10)	— BRB intervention changes expression of 41 colonic mucosa, 40 liver and 34 faecal metabolites — BRB intervention decreases 34 lipid metabolites in colonic mucosa — epigenetic mechanisms not investigated	[54]
black raspberries (BRBs)	gut microbiota study in F344 rats (*n* = 8 per group) for six weeks on — control diet — control diet supplemented with 5% BRBs — control diet supplemented with 0.2% BRB anthocyanin fraction — control diet supplemented with 2.25% BRB residue fraction	— distinct time-dependent changes in gut microbial composition by BRBs or fractions — epigenetic mechanisms not investigated	[55]
**pomegranate extract (PE)** PE-1: 2 mg g^−1^ punicalin, 72 mg g^−1^ punicalagin, 294 mg g^−1^ ellagic acid derivatives PE-2: 5.4 mg g^−1^ punicalin, 155 mg g^−1^ punicalagin, 28 mg g^−1^ ellagic acid derivatives	randomized, double-blind, controlled trial with a daily dose of 900 mg PE-1 or PE-2 for 5–35 days in 35 CRC patients versus 10 control patients	— miRNA levels mostly altered due to the surgical procedure — ellagitannin metabolite levels do not correlate with miRNA expression	[56]
**cruciferous vegetables containing glucosinolates** e.g. broccoli sprouts containing glucoraphanin metabolized to sulforaphane (SFN)	— C57BL/6 J+/+ mice, single oral dose of 15 µmol SFN — C57BL/6 J+/+ and *Apc*^*Min*/+^ mice, 10-week intervention with AIN93G diet supplemented with 443 mgSFN/kg diet (∼ 6 µmol SFN/mouse/day) — human pilot study (*n* = 3), single dose of glucosinolate-rich broccoli sprouts (68 g, containing∼220 µmol glucoraphanin as main glucosinolates [57]), plasma concentration was not assessed	— inhibition of HDAC activity and induction of histone hyperacetylation in mouse colonic mucosa 6 h after dosing — histone hyperacetylation in ileum, colon, prostate and PBMCs after long-term intervention in wild-type mice — in *Apc*^*Min*/+^ mice, prevention of intestinal polyps by approximately 50% and histone hyperacetylation in adjacent normal and tumour tissue — transient HDAC inhibition and histone hyperacetylation in human PBMCs after 3 and 6 h	[57–59]
**dietary fibre** **SCFAs,** e.g. butyrate	— germ-free BALB/c mice colonized with butyrate-producing, mutant, or non-butyrate-producing bacteria (*n* > 10 per group) on low-fibre (2% cellulose), high-fibre (2% cellulose plus 6% fructo-oligosaccharide/inulin) or tributyrin diet (2% cellulose plus 6% tributyrin). — induction of colon carcinogenesis by azoxymethane (AOM, 5 injections)/dextran sodium sulfate (DSS, 3 cycles)	— reduction of tumour burden and increased histone acetylation especially in combination of high-fiber diet and butyrate-producing bacteria, or with butyrate diet in the absence of butyrate-producing bacteria — reduced effects in mice inoculated with mutant bacteria	[60]
**long-chain omega 3 fatty acids**	administration of ALA (1%) with or without *Bifidobacterium breve* (daily dose of 109 microorganisms) to mice (*n* = 8) for eight weeks	— supplementation with *B. breve* in combination with ALA significantly increases liver and brain EPA concentrations — epigenetic mechanisms were not investigated	[61]
long-chain omega 3 fatty acids	AOM-induced colon carcinogenesis in Sprague–Dawley rats, 2 × 2×2 factorial design with *n* = 6 per group — ω3 versus *ω*6 long-chain fatty acids in combination with cellulose or pectin and treatment with the colon carcinogen AOM or saline — diets contain 6 g pectin or cellulose/100 g and either 11.5 g fish oil + 3.5 g corn oil /100 g or 15 g corn oil /100 g — two weekly injections of AOM — sacrifice 10 or 34 weeks after the first injection	— fish oil in combination with pectin is most effective in reducing the number of differentially expressed miRNA in colon (after 10 weeks) and tumour multiplicity (after 34 weeks) — *let-7d, miR-15b, miR-107, miR-191, miR-324* are selectively modulated by fish oil exposure	[62]

### (a) Folate and B-vitamins

Folic acid and other B-vitamins are important cofactors in ‘one carbon metabolism’ to generate SAM for methylation reactions [32]. Dietary sources of folate include green leafy vegetables, asparagus, pulses, nuts, cruciferous vegetables, avocado, papaya, etc. In a dietary intervention study with postmenopausal women, altering plasma levels of folate directly influenced lymphocyte DNA methylation levels [33]. The gut microbiota is also involved in the metabolism of folate and B-vitamins [63]. Selected bacteria are able to synthesize folic acid from pteridine precursors and *p*-aminobenzoic acid [34]. Folate deficiency after antibiotic use indicates that colonic folate production can be significant [63]. In addition to folate, the gut microbiota provides a variety of dietary energy metabolites, such as ATP, NAD^+^ and acetyl-CoA, which are used as cofactors by epigenetic enzymes [35,64].

### (b) Dietary polyphenols

Polyphenols from various sources, including green tea, black raspberries (BRBs), red wine, coffee, apples, isoflavones from soya, curcumin from curry and others, have been reported to affect epigenetic mechanisms *in vitro* in enzymatic reactions and in cell culture (reviewed in [26,27,65]). Studies in animal models for cancer prevention or dietary interventions in humans are limited.

#### (i) Isoflavones from soya

Isoflavones, such as genistein and daidzein, are cancer-preventive phytochemicals with anti-/oestrogenic activity found in soya and other legumes. Epidemiological studies suggest that populations following a typical Asian diet rich in soya products have a reduced risk for hormone-dependent cancers [28]. *S*-(–)equol, a microbial metabolite of daidzein, has higher bioavailability and oestrogenicity than daidzein. Metabolomic investigations have shown that approximately one-third of the Western population and up to 65% of the Asian population is able to produce *S*-(−)equol [66,67]. Variability in gut microbiota composition or human gene polymorphisms, for example, in hormone receptors or hormone binding proteins, may explain individual variability observed in clinical studies investigating biological effects of isoflavones [67]. During the past decade, evidence is accumulating that soya isoflavones including the metabolite *S*-(−)equol affect epigenetic enzymes that write, read or erase epigenetic marks, and subsequently modulate gene expression to counteract the ‘hallmarks of cancer’ (review in [28]). In the few studies performed in humans, gut microbial metabolism has not been systematically taken into consideration, and its influence on epigenetic endpoints should be addressed in future investigations. In addition, pre-clinical models should be carefully chosen to reflect human *in vivo* conditions. For example, rodents very efficiently convert daidzein into *S*-(−)equol [68]. The outcome of investigations on soya in rodent models might, therefore, not be generally informative for human populations.

#### (ii) Green tea catechins

With respect to the modulation of epigenetic mechanisms, green tea catechins (GTCs), with (−)-epigallocatechin gallate (EGCG) as the major catechin, represent one of the best investigated groups of polyphenols. *In vitro*, EGCG was shown to inhibit the activity and expression of DNMTs and to demethylate and re-express genes involved in cell-cycle control (*p16, p21*), cell signalling (RARβ), WNT-signalling (WIF-1), DNA repair (MGMT, hMLH1) and apoptosis (DAPK) (review in [65]).

Lee *et al*. suggested that biotransformation of catechins by Phase II metabolic enzymes might influence the activity of epigenetic enzymes. Catechin metabolism by the catechol-*O*-methyltransferase (COMT) leads to consumption of SAM, which is then less available for the catalytic activity of DNA and histone methyltransferases. On the other hand, methylation reactions by COMT produce *S*-adenosyl-l-homocysteine (SAH), which is a negative feedback inhibitor of methyltransferases [69]. Both depletion of SAM as well as elevated levels of SAH might result in reduced levels of DNA or histone methylation, which might consequentially influence gene expression.

Dietary polyphenols also undergo gut microbial metabolism. The microbial degradation of catechins such as epicatechin (EC) by cleavage of the *O*-heterocycle and dehydroxylation results in the formation of phenolic acids [36,38]. In an enzymatic *in vitro* assay of DNMT activity, protocatechuic acid (at 20 and 40 µM, respectively) inhibited enzyme activity by 60–80% [39]. Waldecker *et al*. investigated microbial metabolites of apple juice extracts on enzymatic HDAC activity. Half-maximal inhibitory concentrations (IC_50_ values) were in the range of 0.19–5.47 mM, exceeding the concentrations of phenolic acids detected in human faecal water [40,41]. Whether these weak *in vitro* inhibitory effects of phenolic acids have physiological relevance needs to be addressed in future investigations *in vivo*.

GTCs have been repeatedly reported to prevent prostate cancer in ‘TRansgenic Adenocarcinoma of Mouse Prostate’ (TRAMP) mice (summary in [65]). Morey Kinney *et al*. used a genome-wide approach to test the influence of GTCs on prostate cancer and DNA methylation in the TRAMP mouse model. Unexpectedly, the intervention with GTCs (0.3% in drinking water) prevented neither prostate cancer growth nor DNA methylation in prostate, liver and gut [50]. Currently, it can only be speculated whether differences in gut microbial populations and alterations in GTC metabolism might explain the observed discrepancies with earlier studies.

#### (iii) Black raspberries

BRBs are a good source for polyphenols including ellagic acid, quercetin glycosides and anthocyanins. Freeze-dried BRBs have been shown to prevent oesophageal and colon cancer in animal models by targeting carcinogen metabolism, cell proliferation, inflammation, angiogenesis and apoptosis, and are well tolerated by humans at daily doses of 45 g [70]. In a small Phase I human dietary intervention study with 20 colon cancer patients, 60 g freeze-dried BRBs per day for a minimum of four weeks led to reduced expression of DNMT1 and promoter demethylation of genes involved in the WNT-signalling pathway in tumour tissue, accompanied by reduced expression of WNT-target proteins such as β-catenin, E-cadherin and Ki67 as proliferation marker [51]. In a subsequent Phase Ib study, the same group investigated whether BRBs might regress rectal polyps in patients with familial adenomatous polyposis (FAP), a genetic disease caused by a mutation of the *APC* gene and characterized by rectal polyps detectable at a young age and high risk for developing colon cancer [52]. Fourteen patients with FAP were treated with BRBs daily for nine months. Seven patients received BRB powder orally plus two BRB suppositories, whereas another seven patients received suppositories together with an oral placebo. Intervention with suppositories was sufficient to reduce polyp number and burden at the end of the study. Three of the 14 patients did not respond to the intervention. In colon tissue of responders, DNMT1 expression (tumours) and *p16* promoter methylation (tumours and adjacent normal tissue) were significantly reduced at the end of the study compared with baseline levels, whereas no changes were detected in the three non-responding patients [52]. The fact that the patients responded differently to the local effects of BRB suppositories indicate that BRB components either are metabolized by the gut microbiota (see *Ellagitannins and urolithins* below) or might influence the gut microbial composition with long-term beneficial effects. These hypotheses were addressed in several rodent studies investigating diets enriched with 5% BRBs in rats [55] and mice [53,54]. In faeces of *Apc^Min/+^* mice, a mouse model of human FAP, BRB intervention for eight weeks significantly increased *Lactobacillus* and *Bacteroidaceae* populations determined by quantitative polymerase chain reaction (qPCR) using population-specific primers, whereas *Bifidobacteriales* and *Ruminococcus* populations were not changed [53]. Similarly, in a study in F-344 rats, six week interventions with diets containing either BRBs, the anthocyanin fraction or the fibre fraction of BRBs, respectively, led to time-dependent alterations in the composition and diversity of gut microbial populations, determined by Roche 454 pyrosequencing of the bacterial 16S gene [55]. Whole BRBs and the fibre fraction increase the abundance of anti-inflammatory bacteria, such as *Akkermansia* and *Desulfovibrio*. Bacteria producing butyrate, a short-chain fatty acid (SCFA) generated by the microbial fermentation of dietary fibre (see *Dietary fibre: short-chain fatty acids*) were increased by whole-BRB-supplemented diet [55]. In wild-type C57BL/6 mice, BRB intervention for eight weeks significantly changed the levels of 41 metabolites in colonic mucosa, 40 metabolites in liver and 34 metabolites in faeces, compared with control diet-fed mice [54]. These studies suggest that alterations in the gut microbiota by dietary BRBs might influence human health. The link between altered BRB metabolite levels and epigenetic gene regulation in colonic tissue still needs to be established.

#### (iv) Ellagitannins and urolithins

Pomegranate, strawberries, blueberries, raspberries, BRBs, nuts and tea are a rich source of ellagitannins [71]. Ellagitannins belong to the polyphenol group of hydrolysable tannins. They are hydrolysed to ellagic acid and further microbially metabolized by decarboxylation and sequential dehydroxylation to urolithins [42,43]. Lactobacilli and *Coriobacteriaceae* (*Gordonibacter*) have been shown to be involved in the metabolism of ellagitannins [72]. Human populations can be stratified into three urolithin-producing groups, depending on the spectrum of urolithin metabolites [73]. Metabotype A (25–80% of the volunteers in different trials) produce only urolithin A conjugates, whereas in metabotype B (10–50%), isourolithin A and/or urolithin B can be detected in addition to urolithin A. Metabotype 0 (5–25%) is not able to produce urolithins, and ellagic acid metabolism stops at the level of urolithin M-6. The three metabotypes were consistently detected, independent of health status, age, gender, body mass index, and amount or type of food source ingested. A higher percentage of metabotype B was associated with gut microbial dysbiosis indicative of chronic diseases in studies on metabolic syndrome and in colorectal cancer (CRC) patients (review in [73]).

Urolithins have a broad spectrum of bioactivities *in vitro* and *in vivo*, including antioxidative, anti-inflammatory, anti-oestrogenic and anti-proliferative activities [42,43,45,73–75]. Several studies have addressed the question whether ellagitannins and urolithins target epigenetic mechanisms, with a focus on miRNAs. Wen *et al*. revealed that incubation of HepG2 cells with the anti-proliferative ellagitannin BJA3121 (50 µg ml^−1^ for 6 h) altered the expression of 25 miRNAs involved in regulation of proliferation and cell differentiation, including 17 upregulated and eight downregulated miRNAs [44]. Gonzáles-Sarrias *et al*. demonstrated that single ellagitannin metabolites or metabolite mixtures (100 µM, 48 h treatment) inhibited cell proliferation and induced cell-cycle arrest and apoptosis in a panel of colon (cancer) cell lines. They identified induction of cyclin-dependent kinase inhibitor 1A (*p21*) as a common target of urolithins and could link *p21* induction with downregulation of onco-miR-224 or upregulation of tumour suppressor miR-215 [45]. Nuñez-Sánchez *et al*. analysed the impact of a daily dose of 900 mg pomegranate extract for 5–35 days before surgery on miRNA expression in colon tissue versus tumour tissue from 35 CRC patients versus 10 control CRC patients in a randomized, double-blind, controlled trial [56]. Surgery led to a general artefactual induction of miRNAs in both normal and tumour tissue. Pomegranate extract intake reversed the surgery-mediated upregulation of various miRNAs and mildly reduced expression of selected miRNAs in tumour tissue compared with normal tissue. However, there was no association between tissue urolithin levels and the observed miRNA expression changes [56]. Similarly, pomegranate extract intervention led to alterations in gene expression, but they were not associated with urolithin levels or metabotypes [76].

In an *in vitro* inflammation model of monocytes stimulated with tumour necrosis factor α (TNFα), 5 µM ellagic acid prevented TNFα-mediated reduction of HDAC activity, whereas ellagic acid and urolithins B and C inhibited the concomitant induction of HAT activity by greater than 50%. The compounds did not directly inhibit HDAC or HAT activity, but might rather target TNFα-stimulated expression changes [46].

In summary, these studies suggest a potential influence of urolithins and other microbial metabolites of polyphenols on epigenetic regulators, and justify a more systematic evaluation of their effects on DNA methylation, histone modifications and miRNA expression to establish a causal relationship.

### (c) Cruciferous vegetables

Cruciferous vegetables are a rich source of glucosinolates as precursors of isothiocyanates (ITCs) and other reactive compounds [77]. ITCs have antimicrobial properties [78] and a broad range of cancer-preventive activities, including inhibition of inflammation and cell proliferation, as well as dose-dependent induction of metabolic detoxification or cell-cycle arrest, apoptosis and autophagy [79,80]. Release of ITCs from glucosinolates is catalysed by the plant-derived thioglucosidase myrosinase. When myrosinase activity is inactivated by cooking, this reaction is dependent on gut bacterial thioglycosidases [81–84]. The diversity of the gut microbiome can, therefore, modulate the bioavailability of ITCs (comprehensive overview in [85]). On the other hand, regular cruciferous vegetable consumption can affect the composition of human gut bacterial communities. In a small randomized, crossover, controlled feeding study involving 17 participants, addition of 14 g kg^−1^ body weight/day cruciferous vegetables for 14 days led to a gut microbiota community shift during the intervention period, with high inter-individual variation both in the baseline microbiota composition and in the response to cruciferous vegetable intake. Also, the authors observed substantial inter-individual variation in ITC excretion after cruciferous vegetable intake [86].

In 2004, Myzak *et al*. first revealed that a cysteine metabolite of sulforaphane, the major ITC released from broccoli sprouts, inhibited HDAC activity *in vitro* [47]. The same group demonstrated HDAC inhibitory activity and histone hyperacetylation in various tissues and intestinal polyps in the *Apc^Min/+^* mouse model. Single ingestion of 68 g fresh broccoli sprouts led to rapid and transient HDAC inhibition in human blood cells [58]. Sulforaphane and other bioactive compounds from cruciferous vegetables also affect additional epigenetic mechanisms (review in [87,88]).

### (d) Dietary fibre: short-chain fatty acids

**Figure 1.**
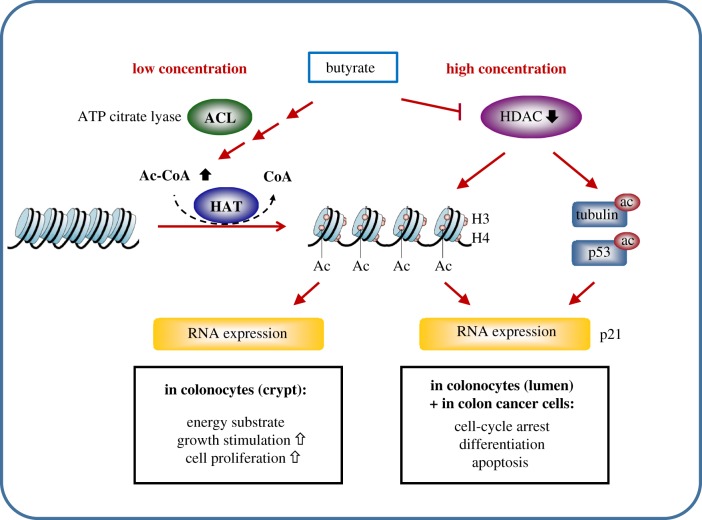
Overview of the concentration-dependent effects of butyrate on histone and non-histone acetylation in human colon. See text for further details. (Online version in colour.)

The fermentation of dietary fibre leads to the formation of SCFAs including acetate, propionate and butyrate. Butyrate serves as a major energy source for intestinal epithelial cells [89,90]. The potential of butyrate to prevent colon carcinogenesis is associated with anti-inflammatory and antioxidative effects, induction of cell differentiation, cell-cycle arrest and apoptosis (comprehensive overview in [91,92]). HDAC inhibitory activity of butyrate was first described almost 40 years ago (summary in [93]). Many of its effects on gene expression and its anti-proliferative activity are related to changes in chromatin structure. Additional cellular targets include acetylation of non-histone proteins, alteration of DNA methylation, inhibition of histone phosphorylation and modulation of intracellular kinase signalling [94]. Butyrate has been reported to increase proliferation in normal colonocytes, in contrast to its effects on colon cancer cells. This ‘butyrate paradox’ has been explained by butyrate concentration-dependent effects in the colon [92]. Donohoe *et al*. proposed that butyrate affects histone acetylation by two distinct mechanisms (figure 1) [48]. In the mammalian colon, two butyrate gradients are formed: the proximal-to-distal luminal gradient arises from bacterial fermentation of fibre and results in a butyrate concentration of about 3.5 mM in the proximal colon, which declines to about 0.5 mM in the distal colon. In addition, a luminal-to-crypt gradient arises because of peristalsis and mucus flow in colonic crypts, with concentrations of 50–800 µM butyrate at the base of the crypt. In colonocytes near the base of the crypt, butyrate at low concentrations is taken up by mitochondria, metabolized to citrate by the tricarboxylic acid (TCA) cycle, and serves as substrate for ATP-citrate lyase (ACL) to generate acetyl-CoA. Acetyl-CoA then stimulates histone acetylation via HAT. At higher doses of butyrate in colonocytes, exceeding the rate of metabolism in the TCA cycle, and in cancer cells that metabolize little butyrate, butyrate accumulates inside nuclei and inhibits HDAC activity, resulting in increased histone acetylation. Although both pathways result in histone hyperacetylation, transcriptomic analyses indicate that different sets of genes are affected. Whereas activation of the acetyl-CoA/HAT pathway induces genes involved in cell proliferation, HDAC inhibition upregulates genes involved in cell-cycle arrest and induction of apoptosis and cell differentiation [48]. Functional relevance of these *in vitro* observations was confirmed in gnotobiotic mouse models colonized with wild-type or mutant strains of a butyrate-producing bacterium to demonstrate that dietary fibre has potent tumour-suppressive effects in a microbiota- and butyrate-dependent manner [60].

### (e) Long-chain omega 3 fatty acids (LC ω3 FA)

Long-chain omega-3 polyunsaturated fatty acids (LC ω3 FAs) are essential fatty acids necessary for human health [95]. α-Linolenic acid (ALA) is a plant-derived LC ω3 FA found in soyabeans, walnuts, dark green leafy vegetables and seed oils. Cold-water fish (fish-oil) are the main source of eicosapentaenoic acid (EPA) and docosahexaenoic acid (DHA). LC ω3 FAs have antioxidant and anti-inflammatory activity, and are incorporated into cellular membranes. Dietary intake of LC ω3 FAs reduces the risk for chronic degenerative diseases including cardiovascular disease, breast and prostate cancer, and depression [96].

LC ω3 FAs have been shown to target epigenetic mechanisms at the level of histone methylation and miRNA expression. Dimri *et al.* identified the histone methyltransferase EZH2 as a target of LC ω3 FAs [49]. EZH2 promotes H3K27 trimethylation at promoter regions to maintain genes in a transcriptional repressive state. Various human breast cancer cell lines were treated with EPA and DHA at 40 and 80 µM concentrations for 3–8 h. Both compounds dose-dependently reduced protein expression of EZH2 by increasing its proteasomal degradation and lowered H3K27me3 levels. As a result, EPA and DHA treatment elevated the levels of the EZH2 targets CDH1 and IGFBP3 and decreased the invasive phenotype [49]. Davidson *et al*. tested the influence of LC ω3 FA intervention on carcinogen-induced rat colon carcinogenesis and concomitant dysregulation of miRNA expression [62]. Rats were fed diets containing fish oil or corn oil in combination with pectin or cellulose. Tumours were induced by two weekly injections with azoxymethane (AOM). Fish-oil intervention significantly reduced the numbers of AOM-induced tumours, especially in combination with pectin. Fish-oil exposure also prevented downregulation of five miRNAs (*let-7d*, *mir-15b*, *miR-107*, *miR-109* and *miR-324–5p*) by AOM treatment, and had the overall strongest reducing effect on the numbers of differentially expressed miRNAs. This study demonstrates that dietary LC ω3 FAs can protect from carcinogen-induced changes in miRNA profiles [62]. Of note, LC ω3 FA formation is affected by the gut microbiota: Wall *et al*. orally co-administered ALA with two strains of *Bifidobacterium breve* to mice and detected elevated levels of EPA in liver and DHA in the brain [61].

## 3. Summary and conclusion

In summary, these examples indicate that the gut microbiota can affect the epigenome in various ways. Long-term dietary choices affect diversity and gene expression of the gut microbiota. On the other hand, the gut microbiota influences bioavailability of dietary agents, and provides energy metabolites as cofactors of epigenetic reactions. Dietary agents may affect the epigenome either directly or through their microbial metabolites. These interactions are best investigated in human intervention studies, as there are differences in intermediary and microbial metabolism between rodents and humans, as exemplified by the ability to produce *S*-(−)equol from the soya component daidzein. Alternatively, inoculation of gnotobiotic animals with human microflora allows investigation of gut microbial metabolites under ‘humanized’ conditions [97].

**Figure 2.**
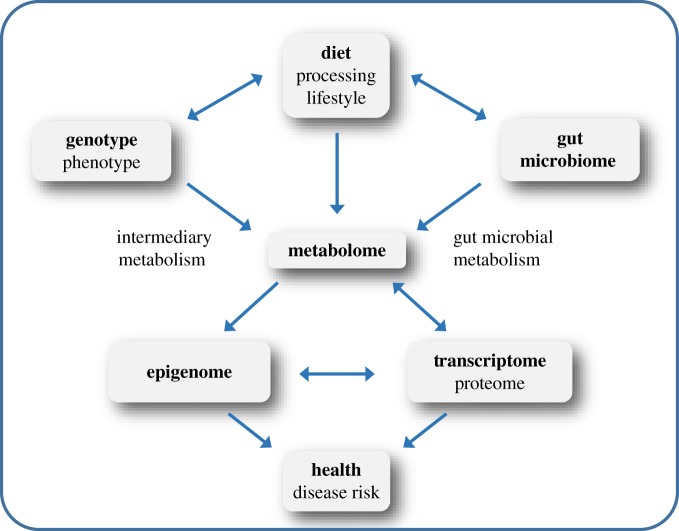
The ‘systems biology’ of nutrition and human health. (Online version in colour.)

Future studies need to integrate information on lifestyle (dietary intake, food processing, information on physical activity, antibiotic use, etc.), genotype, gut microbiome composition and metabolome with genome-wide information on the epigenome and gene/protein expression to fully understand, in a ‘systems biology’ approach, interactions within the system and how to influence them in the direction of improved human health (figure 2). This ambitious goal can only be reached in large interdisciplinary research projects, combining the expertise of food technologists, nutritionists, food chemists, molecular biologists, epigeneticists, clinicians, nutritional epidemiologists, bioinformaticians and statisticians to achieve an integrated view on the influence of diet on human health. Irrespective of promising reports, a causal relationship between modulation of epigenetic mechanisms and prevention of chronic diseases still needs to be established for dietary constituents as well as for their metabolites.

## Data accessibility

This article has no additional data.
